# Phase Ib study of intratumoral talimogene laherparepvec (T-VEC) in combination with chemotherapy or endocrine therapy in patients with advanced HER2-negative breast cancer

**DOI:** 10.1038/s41523-025-00842-8

**Published:** 2025-11-21

**Authors:** Laura A. Huppert, Amelia S. Gliwa, Madeline Tait, Laura Quintal, Stephanie Starzinski, Alexander Cheung, Mark Moasser, Melanie Majure, Michelle Melisko, Pamela Munster, Hope S. Rugo, Michael Campbell, Lawrence Fong, A. Jo Chien

**Affiliations:** 1 University of California, San Francisco Helen Diller Family Comprehensive Cancer Center, San Francisco, CA USA; 2 City of Hope, Duarte, CA USA; 3 Fred Hutchinson Cancer Center, Seattle, WA USA

**Keywords:** Breast cancer, Breast cancer

## Abstract

Talimogene laherparepvec (T-VEC) is an oncolytic virus that is hypothesized to enhance responses to systemic therapy. This Phase 1b trial evaluated the safety and efficacy of intratumoral T-VEC plus chemotherapy (CT) or endocrine therapy (ET) for patients with hormone receptor positive (HR + )/HER2- and triple negative (TN) advanced breast cancer (ABC) with injectable locoregional/chest wall disease. The primary endpoint was safety/tolerability. Secondary endpoints were objective response rate by irRECIST 1.1 and clinical assessment of local response. 19 patients enrolled (9 HR + /HER2-; 10 TN; median two lines of prior CT). Intratumoral T-VEC was administered with the following partners: gemcitabine/carboplatin (*n* = 8), nab-paclitaxel (*n* = 7), paclitaxel (*n* = 2), or ET (*n* = 2). Eight patients in the T-VEC + gemcitabine/carboplatin arm were formally evaluated for dose limiting toxicities (DLTs) based on pre-specified protocol criteria, and one DLT (grade 3 neutropenia leading to carboplatin dose reduction) was identified. Response per irRECIST 1.1 was evaluated in 16 patients: partial response (*n* = 2, 12.5%), stable disease (*n* = 7, 43.8%), progressive disease (*n* = 7, 43.8%). Patients with higher pre-treatment tumor infiltrating lymphocytes (TILs) were more likely to respond, and clinical responders had induction of Ki-67 in multiple peripheral myeloid populations. In conclusion, the addition of intratumoral T-VEC to CT or ET was safe in patients with ABC and injectable locoregional disease, supporting the continued investigation of direct intratumoral immunomodulatory strategies that can enhance local and systemic immune responses. NCT03554044.

## Introduction

Immune recognition of tumor antigens and cytotoxic responses plays an important role in cancer pathogenesis, so agents that enhance the immune response have revolutionized cancer therapy. In breast cancer, immunotherapy with the intravenous PD-1 inhibitor pembrolizumab is approved for early-stage and metastatic triple-negative breast cancer (TNBC)^1,2^, but there is significant interest in being able to harness the immune system to improve the efficacy of systemic therapies across breast cancer subtypes. However, systemic immunotherapy can lead to immune-related adverse events (IRAEs) that can sometimes be severe or life-threatening. Therefore, it is appealing to consider the use of intratumoral therapies to enhance the immune response both locally and systemically (via the abscopal effect), possibly with fewer systemic toxicities. It is also possible that intratumoral therapies can help make “cold” tumors “hot”, i.e., specifically enhance the immunogenicity of less-immunogenic breast cancer subtypes via local tumor cell death and antigen release, which can trigger a systemic immune response.

One intratumoral therapy of interest is Talimogene laherparepvec (T-VEC), which is a genetically modified herpes simplex 1 virus (HSV-1) with deletion of genes ICP34.5 and ICP47 and insertion of granulocyte-macrophage-colony stimulating factor (GM-CSF)^3,4^. These modifications provide tumor specificity, enhance viral replication, increase antigen presentation in infected tumor cells, and induce an anti-tumor immune response. Not only does T-VEC directly kill tumor cells through cell lysis, but prior studies have suggested that it can initiate a systemic antitumor immune response via an abscopal effect^5,6^. T-VEC is currently FDA-approved for the treatment of unresectable cutaneous, subcutaneous, and nodal melanoma based on the results of the pivotal phase III OPTiM study, which demonstrated superior durable response rates with T-VEC compared to subcutaneous GM-CSF in patients with stage IIIB/C or stage IV melanoma^5,6^.

In breast cancer, intratumoral T-VEC was initially studied as monotherapy for ABC in a small phase II study, and it did not demonstrate adequate response and disease control, with 0/9 patients with partial or complete responses^7^. In the early-stage setting, phase I and subsequent phase II studies demonstrated the safety of neoadjuvant intratumoral T-VEC + weekly paclitaxel for 12 weeks, followed by doxorubicin/cyclophosphamide in patients with stage II-III TNBC^8,9^. In the phase II study, the RCB 0 rate was 45.9% and the RCB 0-1 rate was 65%, meeting the study’s primary endpoint with a manageable toxicity profile. While it is not possible to evaluate the activity of T-VEC vs. chemotherapy in this single-arm study, correlative analyses demonstrated evidence of early immune response gene activation and immune cell infiltration after six weeks of intratumoral T-VEC therapy^9^. These data support further evaluation of T-VEC in combination with chemotherapy in ABC.

A clinically applicable way to study T-VEC in ABC is via injection of locoregional or chest wall disease. Approximately 5–10% of patients undergoing mastectomy and 10-15% of patients undergoing breast conserving surgery for operable breast cancer will have a chest wall or regional nodal recurrence within 10 years^10,11^. Chest wall disease is not only associated with a poor prognosis, but also with significant morbidity and is a major quality of life issue due to inevitable complications related to wound control (e.g., pain, bleeding, infection, odor, lymphedema)^12^. Chest wall disease can be difficult to treat and is often resistant to standard locoregional and systemic therapies. The role of surgery is controversial - one study demonstrated that radical surgical resection (if technically feasible) did not improve five-year survival^12^, whereas another study suggested possible benefit from radical surgery, although often with significant morbidity^13^. In the setting of prior radiation therapy, chest wall re-irradiation alone can result in locoregional response, but few patients have long-term local control^14^. Hyperthermia in combination with radiation improved rates of complete response^15,16^, but has not demonstrated an overall survival benefit. There are few studies and limited data evaluating the efficacy of chemotherapy in chest wall disease^17^. Ongoing studies are evaluating novel combination strategies of topical and systemic immunomodulatory agents in combination with chemotherapy for chest wall disease. For example, in a small single-arm phase II study of 15 patients, patients treated with nab-paclitaxel plus topical imiquimod cream, a toll-like receptor-7 agonist, yielded a response rate of 72%, but the duration of response was quite limited^18^. An ongoing study TBCRC-044 is evaluating the efficacy of carboplatin +/− pembrolizumab in patients with chest wall disease^19^. Therefore, despite efforts to better control chest wall disease in patients with ABC with multi-modality therapy, it remains an area of ongoing unmet clinical need.

In this phase 1b study, we evaluated the combination of intratumoral T-VEC with CT or ET in patients with HR + /HER2- and TN ABC with injectable locoregional/chest wall disease. In particular, our goals were to assess: (1) the safety of intratumoral T-VEC in combination with non-taxane chemotherapy; (2) the activity of T-VEC in combination with CT or ET in patients with ABC and locoregional/chest wall disease; (3) the local and systemic T-VEC-associated immune response in both HR + /HER2- and TN tumors.

## Results

### Patient characteristics

Between 2/5/20 and 9/21/22, 19 patients enrolled in this study, including 9 patients with HR + /HER2- ABC (47.4%) and 10 patients with TN ABC (52.6%). Baseline patient characteristics are summarized in Table 1. All patients were female (*n* = 19, 100%) with a median age of 51.6 years (range 33–70 years). Most patients were white (*n* = 13, 68.4%) and non-Hispanic (*n* = 16, 84.2%). Most patients had ductal histology (*n* = 17, 94.7%). There was a high proportion of patients with a history of de novo metastatic disease (*n* = 7, 36.8%), particularly among patients with HR + /HER2- disease (5/9, 55.6%). Most patients had visceral disease (*n* = 14, 73.7%). Patients with HR + /HER2- ABC had a median of two prior lines of endocrine therapy (range 0–6) and two prior lines of chemotherapy (range 0–4) for advanced disease. Patients with TN ABC had a median of two prior lines of chemotherapy (range 1–4), and most had received prior immunotherapy (*n* = 6, 60.0%) for advanced disease.

**Table 1:** Patient characteristics, prior treatment history, and study therapy

Demographic Information	Overall (*n* = 19) n (%)	HR + /HER2- (*n* = 9) n (%)	TNBC (*n* = 10) n (%)
**Sex**			
Female	19 (100.0%)	9 (100.0%)	10 (100.0%)
Male	0 (0.0%)	0 (0.0%)	0 (0.0%)
**Age**			
Median, yrs (range)	51.6 (33–70)	53.2 (33–70)	51.1 (37–69)
**Ethnicity**			
Non-Hispanic	16 (84.2%)	7 (77.8%)	9 (90.0%)
Hispanic or Latino	3 (15.8%)	2 (22.2%)	1 (10.0%)
**Race**			
White	13 (68.4%)	6 (66.7%)	7 (70.0%)
Asian	2 (10.5%)	1 (11.1%)	1 (10.0%)
Black or African American	1 (5.3%)	0 (0.0%)	1 (10.0%)
Other	3 (15.8%)	2 (22.2%)	1 (10.0%)
**Tumor characteristics and sites of disease**			
**Tumor histology**			
Ductal	17 (94.7%)	8 (88.9%)	9 (90.0%)
Mixed ductal and lobular	1 (5.3%)	1 (11.1%)	0 (0.0%)
Adenoid cystic carcinoma	1 (5.3%)	0 (0.0%)	1 (10.0%)
**De novo metastatic disease**			
Yes	7 (36.8%)	5 (55.6%)	2 (20.0%)
No	12 (63.2%)	4 (44.4%)	8 (80.0%)
**Sites of disease**			
Bone	13 (68.4%)	8 (88.9%)	5 (50.0%)
Lymph node	18 (94.7%)	8 (88.9%)	10 (100.0%)
Skin/chest wall	12 (63.2%)	7 (77.8%)	5 (50.0%)
Breast/soft tissue	12 (63.2%)	6 (66.7%)	6 (60.0%)
Liver	7 (36.8%)	6 (66.7%)	1 (10.0%)
Lung	9 (47.4%)	4 (44.4%)	5 (50.0%)
Brain	2 (10.5%)	1 (11.1%)	1 (10.0%)
**Visceral disease**			
Yes	14 (73.7%)	9 (100.0%)	5 (50.0%)
No	5 (26.3%)	0 (0.0%)	5 (50.0%)
**Treatment history for MBC**			
**Prior immunotherapy for MBC**			
Yes	7 (36.8%)	1 (11.1%)	6 (60.0%)
No	12 (63.2%)	8 (88.9%)	4 (40.0%)
**Prior lines of therapy for MBC**			
Median lines of chemotherapy for MBC, n (range)	2 (0–6)	2 (0–6)	2 (1–4)
Median lines of endocrine therapy for MBC, n (range)	2 (0–4)	2 (0–4)	n/a
Median total lines of therapy for MBC, n (range)	4 (0–8)	4 (0–8)	2 (1–5)
**Study Treatment**			
**T-VEC exposure**			
Median number of T-VEC doses received, weeks (range)	4 (1–14)	3 (1–14)	4 (2–9)
Site(s) of T-VEC injection			
Fungating breast or chest wall mass	7 (36.8%)	4 (44.4%)	3 (30.0%)
Breast tumor	7 (36.8%)	3 (33.3%)	4 (40.0%)
Subcutaneous nodule	5 (26.3%)	2 (22.2%)	3 (30.0%)
**Systemic treatment partner with T-VEC**			
Gemcitabine/carboplatin	8 (42.1%)	3 (33.3%)	5 (50.0%)
Nab-paclitaxel	7 (36.8%)	3 (33.3%)	4 (40.0%)
Paclitaxel	2 (10.5%)	1 (11.1%)	1 (10.0%)
Endocrine therapy (tamoxifen or fulvestrant)	2 (10.5%)	2 (22.2%)	0 (0.0%)

*yrs* years, *HR* *+* */HER2-* hormone-receptor positive, human epidermal growth factor receptor-2 negative, *TNBC* triple negative breast cancer, *MBC* metastatic breast cancer.

### Study treatment

Study treatment is summarized in Table 1. All 19 patients (100.0%) received at least one dose of T-VEC. The median number of T-VEC doses delivered was 4 (range 1–14). The patient’s primary type of locoregional disease where T-VEC was injected included: fungating breast or chest wall mass(es) (*n* = 7, 36.8%), non-fungating breast mass(es) (*n* = 7, 36.8%), or subcutaneous nodule(s) (*n* = 5, 26.3%). Patients received T-VEC with the following treatment partners at the discretion of the treating provider: gemcitabine/carboplatin (G/C, *n* = 8, 42.1%), nab-paclitaxel (nab-P, *n* = 7, 36.8%), paclitaxel (P, *n* = 2, 10.5%), or endocrine therapy including tamoxifen or fulvestrant (ET, *n* = 2, 10.5%).

### Safety and toxicity

The combination of T-VEC with G/C was evaluated for DLTs per the pre-defined study protocol. Of the 8 patients receiving G/C and T-VEC, 5 patients (63%) received 4 mL (2 mg) T-VEC with each intratumoral injection and 3 patients (37%) received 2 mL (1 mg) T-VEC with each intratumoral injection. Among these eight patients, there was one DLT (grade 3 neutropenia leading to subsequent carboplatin dose reduction on C1D8); the chemotherapy dose reduction during cycle 1 was the defined reason for DLT per protocol. While not a pre-defined endpoint per protocol, there were nine patients treated with paclitaxel or nab-paclitaxel and TVEC and 1/9 (11%) met criteria for DLT due to a paclitaxel-related infusion reaction, which was unrelated to TVEC administration.

The most common treatment-emergent adverse events (AEs) in all patients are summarized in Table 2 and data by treatment arm are provided in Supplementary Table S4. The most common any-grade treatment-emergent AEs included: fever in the first 24 h after T-VEC injection (*n* = 14, 73.7%), fatigue (*n* = 14, 73.7%), nausea (*n* = 10, 52.6%), and chills (*n* = 8, 42.1%). 9 patients (47.4%) experienced a grade 3/4 AEs, including neutropenia (*n* = 6, 31.6%), thrombocytopenia (*n* = 2, 10.5%), anemia (*n* = 1, 5.3%), and injection site skin ulceration (*n* = 1, 5.3%). Four patients had injection site skin ulceration in total: 3 with grade 1 ulceration (15.8%) and 1 with grade 3 ulceration (5.3%); all four patients held T-VEC due to ulceration and continued systemic treatment, and one patient subsequently re-initiated T-VEC at a different injection site. For all four patients, the injected site was classified as a fungating breast/chest wall mass, and ulceration occurred during cycle 2 in all cases. T-VEC was held at the site of ulceration for all patients, and CT or ET was continued; one patient had T-VEC injected at another non-ulcerated site of disease. Ulcerations persisted until the end of study therapy for three of the four patients. One patient with a history of baseline HSV infection experienced a grade 2 localized HSV outbreak on the buttock during the study, which was treated with topical acyclovir and was unrelated to study therapy.

**Table 2:** Treatment-related adverse events

Treatment-related adverse event (*n* = 19)	Any grade, n (%)	Grade 3-4, n (%)
Injection site pain	7 (36.8%)	0 (0%)
Injection site erythema/redness	2 (10.5%)	0 (0%)
Injection site skin ulceration	4 (21.1%)	1 (5.3%)
Chills	8 (42.1%)	0 (0%)
Fever	14 (73.7%)	0 (0%)
Body aches/myalgias	4 (21.1%)	0 (0%)
Fatigue	14 (73.7%)	0 (0%)
Nausea	10 (52.6%)	0 (0%)
Emesis	3 (15.8%)	0 (0%)
Anorexia	4 (21.1%)	0 (0%)
Neuropathy	5 (26.3%)	0 (0%)
Dysgeusia	4 (21.1%)	0 (0%)
Diarrhea	3 (15.8%)	0 (0%)
Infusion reaction	3 (15.8%)	0 (0%)
Anemia	3 (15.8%)	1 (5.3%)
Thrombocytopenia	6 (31.6%)	2 (10.5%)
Neutrophil count decreased	7 (36.8%)	6 (31.6%)

Shown are the most frequent treatment-related adverse events by CTCAE v 4.0, including any treatment-related adverse event present in >10% patients and/or any grade 3 or greater event. No grade 5 events occurred.

*n* number.

### Clinical responses

**Fig. 1:**
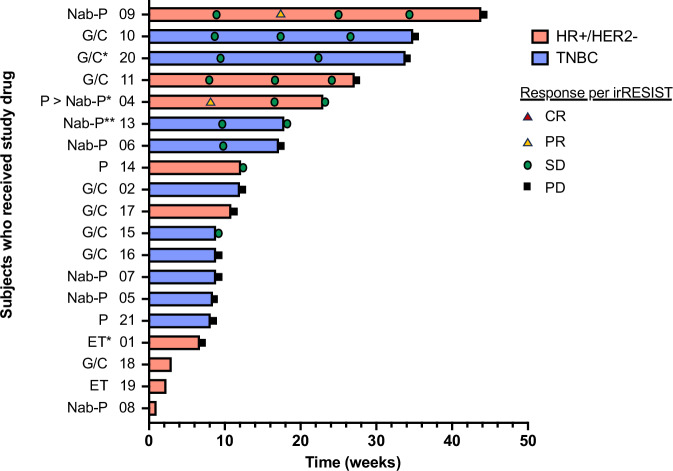
Efficacy of intratumoral T-VEC + systemic therapy. Swimmer’s plot of duration of therapy and responses per irRESIST. Red bars indicate patients with HR + /HER2- disease, and blue bars indicate patients with TNBC. The systemic treatment partner is shown at left, with abbreviations as follows: G/C gemcitabine/carboplatin, nab-p nab-paclitaxel, p paclitaxel, ET endocrine therapy; * = T-VEC stopped early due to local site ulceration; systemic therapy continued until progression. ** = T-VEC stopped due to ulceration but then restarted at a different injection site. The patient ID number (PID) is also shown at left next to the systemic treatment partner. Responses per irRESIST are shown using the following symbols: Red triangle, complete response (CR); Yellow triangle, partial response (PR); Green circle, stable disease (SD), Black square, progressive disease (PD). PIDs 18, 19, and 08 at the bottom had rapid clinical progression prior to the first re-staging scan so irRESIST not available.

Clinical responses to intratumoral T-VEC + chemotherapy or endocrine therapy are summarized in Fig. 1, Table 3, and Supplementary Table S5. Of the 19 patients, 16 patients were evaluable by irRECIST, and 3 were non-evaluable due to rapid clinical progression after ≤1 dose of T-VEC. Of the 16 evaluable patients by irRECIST, there were 2 patients with partial responses (PR) (12.5%), 7 patients with stable disease (SD) (43.8%), and 7 patients with progressive disease (PD) (43.8%). The ORR was 12.5% and the clinical benefit rate (CBR) was 56.3%. The median duration of time on treatment was 12.1 weeks (range 6.7–43.9 weeks).

**Table 3:** Best response by irRECIST and best local response by clinical assessment

Best response by irRECIST^a^ (*n* = 16)	Overall (*n* = 16) n (%)	HR + /HER2- (*n* = 6) n (%)	TNBC (*n* = 10) n (%)
CR	0 (0.0%)	0 (0.0%)	0 (0.0%)
PR	2 (12.5%)	2 (33.3%)	0 (0.0%)
SD	7 (43.8%)	2 (33.3%)	5 (50.0%)
PD	7 (43.8%)	2 (33.3%)	5 (50.0%)
ORR (CR + PR)	2/16 (12.5%)	2/6 (33.3%)	0/10 (0.0%)
CBR (CR + PR + SD)	9/16 (56.3%)	4/6 (66.6%)	5/10 (50.0%)
Duration of time on treatment, weeks (range)	12.1 (6.7–43.9)	17.6 (6.7–43.9)	10.5 (8.1–34.9)
**Best local response by clinical assessment of skin/chest wall/breast disease**^**b**^ **(n** = **18)**	**Overall (n** = **18)** n (%)	**HR** + **/HER2- (n** = **8)** n (%)	**TNBC (n** = **10)** n (%)
CR	0 (0.0%)	0 (0.0%)	0 (0.0%)
PR	8 (44.4%)	3 (37.5%)	5 (50.0%)
SD	7 (38.9%)	3 (37.5%)	4 (40.0%)
PD	3 (16.7%)	2 (25.0%)	1 (10.0%)
ORR (CR + PR)	8/18 (44.4%)	3/8 (37.5%)	5/10 (50.0%)
CBR (CR + PR + SD)	15/18 (83.3%)	6/8 (75.0%)	9/10 (90.0%)

^a^irRESIST was used for evaluation per the pre-specified analysis plan. Three patients had rapid clinical progression before the first restaging scan, and thus they are not included in irRESIST efficacy results in Table 3. Of note, RESIST was also evaluated, and the best responses were the same for all patients except two instances (PID 13, 15) where the response was categorized as SD by irRESIST but PD by RESIST. Duration of time on treatment was defined as C1D1 to date of progression or treatment discontinuation for any reason.

^b^Best local response was assessed clinically by the treating physician and verified/validated by review of clinical photos and disease measurement.

*n* number, *CR* complete response, *PR* partial response, *SD* stable disease, *PD* progressive disease, *ORR* objective response rates, *CBR* clinical benefit rate, *PFS* progression-free survival, *HR* *+* */HER2−* hormone-receptor positive, human epidermal growth factor receptor-2 negative, *TNBC* triple negative breast cancer.

Of the six patients with HR + /HER2− disease evaluable by irRECIST, the ORR was 33.3%, the CBR was 66.6%, and the median duration of time on treatment was 17.6 weeks (range 6.7–43.9 weeks).

All ten patients with TNBC were evaluable by irRECIST, there were no patients who achieved a PR or CR. The CBR was 50.0%, and the median duration of time on treatment was 10.5 weeks (range 8.1–34.9 weeks). There was no association between PDL1 status and response (Supplementary Table S5).

In addition to the evaluation of systemic response by irRECIST, disease response at the local site of T-VEC injection was clinically evaluated by the treating provider’s measurement and assessment in 18 patients (see example Supplementary Fig. S2). The best local clinical responses were as follows: CR (*n* = 0, 0.0%), PR (*n* = 8, 44.4%), SD (*n* = 7, 38.9%), PD (*n* = 3, 16.7%). Of note, all four patients with ulceration had a clinical response locally; 3/4 had systemic response but 1/4 had systemic progression.

### Immune response assessment in blood

**Fig. 2:**
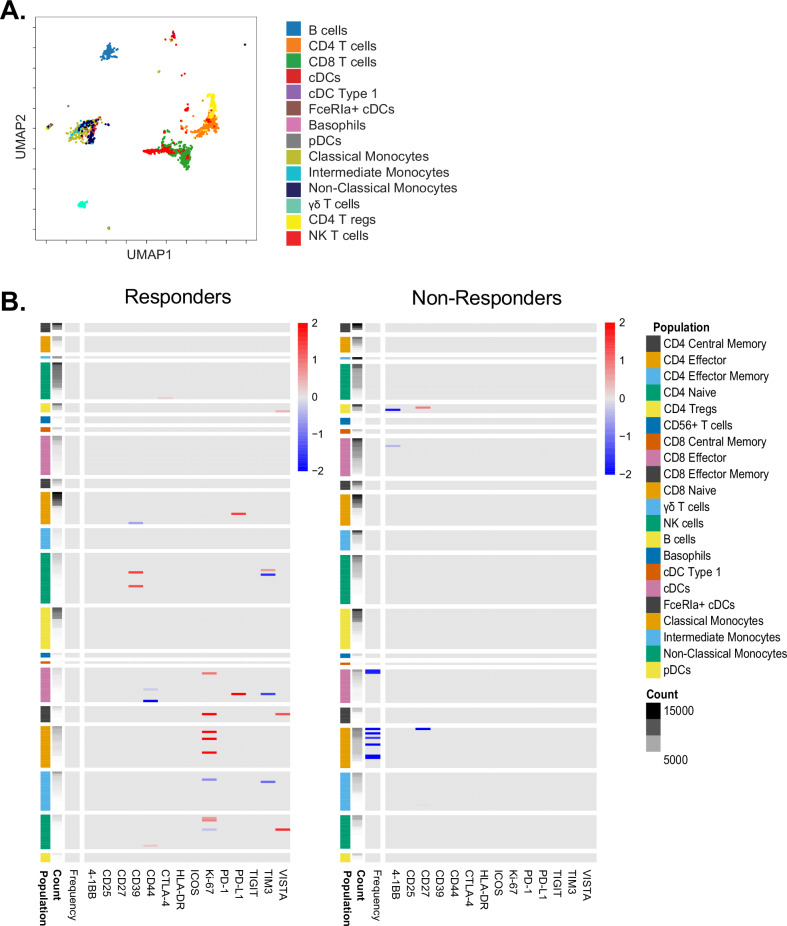
Flow cytometry analysis of circulating immune cells. Peripheral blood mononuclear cells (PBMCs) were stained and assessed by mass cytometry. **A** Immune cells were annotated based upon phenotypic gating markers. **B** Statistical scaffold was then applied to assess for changes between baseline and C2D15 timepoints in responders (left panel) and in non-responders (right panel). Heatmaps summarizing log_2_ fold changes resulting from statistical scaffold analysis of cell cluster frequency (left-most column and functional markers (4-1BB, CD25, CD27, CD39, CTLA-4, HLA-DR, ICOS, Ki-67, PD-1, PD-L1, TIGIT, TIM3, and VISTA) are shown comparing baseline vs C2D15. Red denotes an increase in the C2D15 timepoint; blue denotes a decrease in the C2D15 timepoint relative to baseline.

To determine whether the treatment modulated circulating immune cell populations, we performed mass cytometry on cryopreserved PBMCs (Fig. 2A). Following cellular annotation (Supplementary Fig. S3A), we performed a Statistical scaffold to compare baseline and C2D15 timepoints (Supplementary Fig. S3B). We found that the treatment led to a decrease in circulating myeloid cells, including dendritic cells and classical monocytes (Supplementary Fig. S4A). Comparing local responders with non-responders by irRESIST, we found that there were minimal differences in circulating immune cells at baseline (Supplementary Fig. S4A). However, when we evaluated treatment-induced changes between baseline and C2D15, we found that responders had an increase in Ki-67 on dendritic cells, classical and non-classical monocytes, while non-responders did not (Fig. 2B). We confirmed these results through manual gating of these populations (Supplementary Fig. S5).

### Immune response assessment in tumor tissue

**Fig. 3:**
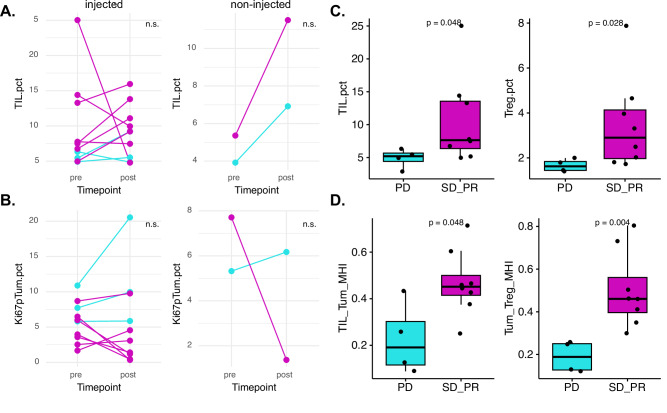
Association of immune infiltrates and proliferating tumor cells with response to T-VEC + systemic therapy. **A**, **B** Tumor-infiltrating lymphocyte (TIL) density (**A**) and proliferating tumor cell density (**B**) are shown for baseline (pre) and C2D15 (post) tumor biopsies. The left side in each panel shows results from injected lesions and the right side shows results from non-injected lesions. Clinical benefit rate by irRECIST is color coded as progressive disease (cyan) and stable disease or partial response (magenta). **C**, **D** TIL and regulatory T cell (Treg) percentages (**C**) as well as spatial proximity (Morisita-Horn Index) of tumor cells with TIL or Tregs (**D**) in pre-treatment injected lesions were significantly associated with clinical benefit rate by irRECIST (PD progressive disease; SD_PR stable disease or partial response). *p*-values from Wilcoxon rank sum test, n.s. indicates not significant.

We also performed multiplex immunofluorescence (mIF) on tumor tissue samples from pre- and post-treatment in both the injected and a neighboring non-injected lesion, if available. Comparing baseline to C2D15 injected biopsies, we observed an increase in tumor-infiltrating lymphocytes (TILs) in 4/7 patients with SD or PR and 2/3 patients with PD (Fig. 3A). mIF data from matched baseline and C2D15 biopsies of non-injected lesions was available for two cases (one PD and one SD) and both showed increases in TILs (Fig. 3A). The percent of proliferating (Ki67^+^) tumor cells in injected lesions decreased in 4/7 patients with SD or PR and increased or remained the same in 3/3 PD cases (Fig. 3B). In the two cases with mIF data from baseline and C2D15 non-injected biopsies, Ki67^+^ tumor cells decreased in the patient with SD but increased in the patient with PD.

We examined immune infiltrates in baseline pre-treatment biopsies for their associations with clinical benefit rate (PD vs SD or PR). Of the 23 cell populations identified by mIF (Supplementary Table S3), levels of TILs and Tregs were significantly higher in responders (SD or PR) vs. non-responders (PD) (Fig. 3C). Example images of low TIL infiltrates in a non-responder vs. high TIL infiltrates in a responder are shown in Supplementary Fig. S6. We next examined spatial relationships between cells in the tumor microenvironment, calculating the Morisita-Horn index for 20 pairs of cell types (Supplementary Table S3), and found proximity scores of tumor cells and TILs, as well as tumor cells and Tregs, were significantly higher in responders vs. non-responders (Fig. 3D).

## Discussion

In this Phase 1b single-arm study, we demonstrated that the addition of intratumoral T-VEC to CT or ET was safe and tolerable in patients with ABC and injectable locoregional disease in the breast, chest wall, or subcutaneous nodules.

The primary objective of this study was to assess the safety of T-VEC in combination with CT or ET. We found that the combination of T-VEC with gemcitabine and carboplatin was safe, and only one DLT (grade 3 neutropenia) was observed. AEs were consistent with prior reports of intratumoral T-VEC use, including fever in the first 24 h, chills, fatigue, and injection site pain^7–9^. Interestingly, four patients in this study had injection site skin ulceration, including three grade 1 events (15.8%) and one grade 3 event (5.3%). It is unclear if this ulceration occurred in response to T-VEC injection or whether this was due to the natural history of the disease. Small numbers do not allow identification of risk factors for injection-related ulceration, but notably, all four cases occurred in fungating breast/chest wall masses, which perhaps may not heal as well with the injection of lidocaine and T-VEC. Of note, ulceration was not a reported treatment-related adverse event in the phase III OPTiM study in patients with stage IIIB/C or stage IV melanoma^5,6^. Further data is needed to better understand this phenomenon, but patients with fungating breast or chest wall should be monitored closely for skin ulceration at the site of injection.

A key secondary endpoint was evaluation of the ORR T-VEC in combination with CT or ET via assessment of systemic response by irRESIST 1.1 and by local response assessment at the site of T-VEC injection. The efficacy data per irRECIST 1.1 were promising: 2 patients had PRs (12.5%), 7 patients had SD (43.8%), and 7 patients had PD (43.8%), although it is not possible to define the additive benefit of T-VEC in this non-randomized Phase 1b trial. Chest wall disease is often non-measurable and not well reflected per standard RECIST criteria; however, it can cause significant morbidity; therefore, local disease control is a meaningful clinical endpoint and was evaluated as an independent endpoint by having treating providers measure and assess clinical local response based on physical exam. We observed a 44% local response rate, so this is a potentially clinically meaningful strategy to improve the morbidity associated with chest wall lesions. Prior work demonstrated that intratumoral T-VEC monotherapy was ineffective in controlling breast cancer chest wall disease (0/9 patients)^7^. Chest wall disease tends to have a chemotherapy-resistant biology and follow an aggressive clinical course^12^, so the favorable local response rates seen in this study are encouraging that T-VEC or other local intratumoral immunomodulatory strategies have the potential to offer meaningful local disease control when used in combination with other systemic therapy strategies. Further work is needed to clarify the specific contribution of intratumoral T-VEC and specifically whether it can enhance local and/or systemic responses.

The addition of pembrolizumab is now standard of care for patients with early-stage TNBC and patients with metastatic TNBC who are PD-L1 positive^1,2^. There are also patients with high-grade early-stage HR + /HER2- breast cancer who may benefit from immunotherapy^20,21^. However, systemic immunotherapy can lead to IRAEs, which can cause significant morbidity and mortality. It is appealing to consider the use of intratumoral therapies to enhance the immune response both locally and systemically (via the abscopal effect), possibly with fewer systemic toxicities. Currently, T-VEC is the only FDA-approved intratumoral oncolytic viral therapy, but other intratumoral therapies are under investigation, including other oncolytic viral therapies such as VV1, Pexa-Vec, and FH10, and non-oncolytic viral therapies such as PV-10 and toll-like receptor 9 agonists^22,23^. For example, VV1 is a vesicular stomatitis virus engineered to express human interferon-beta and HIFN-beta, which increases anti-tumoral immune response^24^. SD101, a TLR9 agonist, has been evaluated intratumorally with neoadjuvant systemic paclitaxel and pembrolizumab in the ISPY trial^25^, which resulted in increased regional lymphadenopathy and immune infiltration^26^, but there was no change in the rate of pathologic complete response (pCR)^25^. This suggests that immune activation does not always translate into improved anti-tumor activity, perhaps due to increased T cell exhaustion. There are also ongoing studies evaluating the role of alternative injection sites, including visceral metastases, for T-VEC^27^ and other intratumoral therapies^23,28^, which may also provide local and abscopal effects. Further work is needed to clarify whether intratumoral therapies can be effectively harnessed to increase the efficacy of systemic therapies, ideally with a reduction of systemic toxicities.

Exploratory correlative analyses using blood and tissue samples were performed to investigate whether a T-VEC injection alters the tumor immune microenvironment and whether a systemic response could be induced. In melanoma, T cell phenotypic characterization of TILs and peripheral blood lymphocytes in patients treated with T-VEC demonstrated increased intratumoral CD8 + T cells, neutrophils, monocytes, and chemokines, as well as increased peripheral CD4 + T cells, CD8 + T cells, and myeloid populations^29,30^. Similarly, in our study, mass cytometry analysis of cryopreserved PBMCs demonstrated changes in circulating immune cell populations after initiation of treatment. Interestingly, we found that clinical responders had increased Ki-67 expression in multiple myeloid populations, which was not observed in non-responders. This induction of proliferating myeloid populations could represent biologic effects of T-VE,C which expresses GM-CSF, a cytokine known to induce myeloid cell expansion, or it could be related to the T-VEC or chemotherapy-induced immune induction. Mass spectrometry of tissue samples from this study is planned. We also performed multiplex immunofluorescence (mIF) to evaluate local responses at the site of T-VEC injection via analysis of pre- and post-treatment paired tissue samples from injected and non-injected lesions. In breast cancer, there is mounting evidence that cancers with TILs are most responsive to chemotherapy and immunotherapy^31,32^. Similarly, pre-treatment levels of TILs and Tregs, as well as their spatial colocalization with tumor cells, were associated with overall clinical benefit rate and could potentially act as biomarkers to predict response to T-VEC therapy. We observed increased TILs in 6/10 evaluable paired injected samples. Similarly, prior work by Soliman et al. evaluating the efficacy of intratumoral T-VEC plus paclitaxel in patients with early-stage TNBC demonstrated increased TILs after 6 weeks of therapy^9^. In the present study, an increase in TILs was seen in 2 patients with evaluable non-injected tissue pre- and post-therapy, suggesting the possibility of the induction of a systemic abscopal effect, although the relative impact of T-VEC vs. the partner chemotherapy/endocrine therapy is difficult to distinguish.

This study has several notable strengths. First, there are few studies that specifically enroll patients with advanced breast cancer and chest wall disease, which is an area of high unmet clinical need. Second, the collection of both injected and non-injected tissue at multiple timepoints during the study provides the ability to analyze responses before and after treatment in both the directly injected and at more distant sites.

This study also has several limitations. First, this was a single-arm study, so it is not possible to determine the impact of intratumoral T-VEC vs. that of chemotherapy or endocrine therapy. Moreover, the inclusion of different systemic therapy partners (gemcitabine/carboplatin, paclitaxel, endocrine therapy) introduced variability, complicating the interpretation of results, although it provided safety data in multiple combinations, which can be used to inform future studies. Second, the translational analyses analyzing both injected and non-injected lesions in each individual patient aimed to determine the potential impact of TVEC (direct and/or abscopal) in each individual patient. However, not all patients had both pre- and on-treatment biopsies of both injected and non-injected sites, limiting the ability to do these comparisons, and it is difficult to assess the impact of the systemic therapy vs. the possible abscopal effect of TVEC. Third, the evaluation of local response assessment was determined by the study investigator, so there may have been some variability and bias in assessment of local treatment response. To try to standardize assessment, we required measurements of each target lesion with each cycle. Despite limitations in local assessment, we still wanted to capture local response as an independent exploratory endpoint as these changes may not be adequately captured by irRECIST criteria, and local response or lack thereof can affect patient quality of life in cases of aggressive chest wall disease. Fourth, three patients were unevaluable for systemic response due to rapid disease progression, emphasizing the aggressive nature of the disease and suggesting a need for earlier intervention and/or more stringent patient selection.

In conclusion, the results from this Phase Ib trial demonstrated that the addition of intratumoral T-VEC to chemotherapy or endocrine therapy is safe and tolerable in patients with ABC and locoregional disease. Patients with fungating breast or chest wall lesions should be monitored closely for skin ulceration at the site of injection. Patients treated with chemotherapy or endocrine therapy plus T-VEC had treatment-induced changes in both local tumor and circulating immune cell populations. These results support the additional clinical development of intralesional anti-tumor strategies in combination with systemic therapy as a novel treatment combination for the treatment of patients with ABC with chest wall disease.

## Methods

### Patients

Male or female patients ≥18 years who had histologically confirmed locally advanced, unresectable, or metastatic HER2-negative breast cancer with at least one locoregional lesion (e.g., breast, chest wall, skin nodules, axillary, or supraclavicular lymph node) that was ≥1 cm and locally accessible for intratumoral injection were eligible to enroll. There were no limits on prior lines of chemotherapy. Eligibility included an Eastern Cooperative Oncology Group performance status (ECOG PS) of 0-1 and adequate organ and bone marrow function. Patients with stable treated brain metastases were eligible to enroll. Exclusion criteria included known active brain metastases and/or leptomeningeal disease, a history of symptomatic autoimmune disease or active autoimmune disease that required systemic treatment in the two weeks prior to enrollment (prednisone 10 mg or greater or equivalent), active herpetic skin lesions, or prior complications of HSV-1 infection (e.g., HSV-associated encephalitis, keratitis). The study was approved by the University of California, San Francisco (UCSF) Committee on Human Research, and written informed consent was obtained from all patients prior to enrollment. Research performed in accordance with the Declaration of Helsinki. This trial was registered at ClinicalTrials.gov (NCT03554044).

### Study design

This was a single-center open label Phase 1b trial. The primary objective was to evaluate the safety and tolerability of T-VEC in combination with chemotherapy (paclitaxel, nab-paclitaxel, or gemcitabine/carboplatin) or endocrine therapy (Supplementary Fig. S1). Safety was previously reported for the combination of T-VEC and weekly paclitaxel^8,9^, so based on pre-specified protocol criteria, only patients enrolled in the G/C + T-VEC arm were formally evaluated for dose-limiting toxicities (DLTs). The maximum tolerated volume (MTV) was defined as the highest T-VEC volume (up to maximum 4 mL) that resulted in ≤1 Dose Limiting Toxicity (DLT) per six patients enrolled in the gemcitabine/carboplatin group during cycle 1, with dose-deescalation and stopping rules applied if more than 1/6 patients had DLT events. Secondary objectives were to evaluate the efficacy of T-VEC in combination with chemotherapy or endocrine therapy using irRECIST version 1.1 criteria, including ORR and response duration, as well as local clinical response to T-VEC injection.

The choice of partner chemotherapy or endocrine therapy was at the discretion of the treating physician. The chemotherapy options included: 1) gemcitabine 1000 mg/m^2^ in combination with carboplatin (AUC 2) on D1 and 8 of a 21-day cycle or 2) weekly nab-paclitaxel 100 mg/m^2^ or paclitaxel 80 mg/m^2^ on D1, 8, +/− 15 of a 21-day cycle. The endocrine therapy options for this protocol included: letrozole, anastrozole, exemestane, tamoxifen, or fulvestrant.

T-VEC was administered by intralesional injection with or without ultrasound guidance (first dose 10^6^ PFU/mL followed by 10^8^ PFU/mL 3 weeks later, then every 2 weeks for 6 weeks, and then every 3 weeks thereafter). The maximum total volume of T-VEC injected on any given treatment day depended on the size/number of injectable sites of disease. Patients with multiple lesions could receive an injection at multiple sites as long as the maximum total volume per treatment day was not exceeded. The maximum T-VEC injection volume per tumor size was as follows: Tumor >5.0 cm = 4.0 mL T-VEC; Tumor >2.5 to 5.0 cm = 2.0 mL T-VEC; Tumor >1.5 to 2.5 cm = 1.0 mL T-VEC; Tumor 1 to 1.5 cm = 0.5 mL T-VEC. The largest and most symptomatic lesions were prioritized and injected with the appropriate volume based on the criteria above. Additional lesions were prioritized from largest to smallest until the maximum volume per treatment day (4 mL) was reached. If there were more than one injectable lesion, at least one lesion was left un-injected for the first two cycles in order to obtain biopsies of treated and untreated lesions.

The DLT evaluation period was one three-week cycle and was only formally evaluated in the gemcitabine/carboplatin group, given prior safety data for combination with weekly paclitaxel^8,9^. Maximum total T-VEC dose/volume per treatment day was not increased beyond 4 mL (which is also the starting dose/volume level) but could be de-escalated if 4 mL was found to be intolerable. DLT events were defined as any one of the following AEs that occurred during the first cycle of study treatment and deemed to be related or possibly related to study treatment: (1) Herpetic events (e.g., serious herpetic events such as encephalitis, encephalomyelitis, or disseminated herpetic infection) or any herpetic event that requires systemic acyclovir or similar anti-viral agent. (2) Non-herpetic dose-limiting toxicity: grade 3 or higher non-herpetic, non-hematologic toxicity, except any grade alopecia. (3) Hematologic dose-limiting toxicity: grade 4 neutropenia lasting 7 or more days or grade 2 neutropenia with fever >38. 5 °C, grade 4 thrombocytopenia ( ≤ 25.0 × 10^9^/L), or grade 3 thrombocytopenia complicated by bleeding and/or requiring platelet or blood transfusion, 4) failure to administer chemotherapy during cycle 1 due to a treatment-related toxicity, T-VEC dose reductions during cycle 1 due to treatment-related toxicity, or the need to delay cycle 2 by more than 21 days due to toxicity.

Patients were continued on study treatment until disease progression as assessed by irRECIST version 1.1, clinical exam, and/or intolerable toxicity. Per protocol, patients were evaluable for response only if they had at least two cycles of study therapy and had their disease re-evaluated. If there were no lesions appropriate for injection due to treatment response or side effects, patients could remain on study and continue systemic therapy without receiving intratumoral T-VEC injections. Patients who wished to discontinue chemotherapy due to toxicity after cycle 3 could remain on T-VEC monotherapy or switch to endocrine therapy (if indicated) and continue receiving T-VEC alone.

### Clinical assessments

Response was evaluated every 9 weeks by CT chest/abdomen/pelvis with bone scan or PET/CT until disease progression based on objective tumor assessments using irRECIST version 1.1 criteria. Clinical evaluation of the breast/chest wall/skin was evaluated by investigator assessment, and lesions were measured each cycle, with overall assessment each cycle per the study investigator. AE severity was graded in accordance with the National Cancer Institute Common Terminology Criteria for Adverse Events (CTCAE version 4.0). Filgrastim or pegylated-filgrastim myeloid growth factor support was allowed at the discretion of the treating physician.

### Mass cytometry

Cryopreserved whole blood peripheral blood mononuclear cells (PBMCs) were obtained at baseline (pre-treatment) and at C2D15 (or at progression if PD happened prior to C2D15). Available cryopreserved PBMCs were thawed, barcoded, and stained with heavy metal-labelled antibodies for analysis by mass cytometry by time-of-flight (CyTOF) (Supplementary Table S1). The samples were then washed and acquired on a mass cytometer (Helios, Fluidigm). Contour plots were generated for CD3^+^ T cells with Cytobank^33^. We used *flowCore* v2.0.1 (https://rdrr.io/bioc/flowCore/) to load the data into R 4.0.2, uwot (https://arxiv.org/abs/1802.03426) to make the UMAP projection, Rphenograph^34^ for clustering, and ggplot2 (https://ggplot2.tidyverse.org) for plotting. The calculation of both the UMAP projection and the clustering were based on the relative staining of CD3, CD4, CD8a, CD11b, CD11c, CD14, CD16, CD19, CD25, CD31, CD33, CD45RA, CD56, CD66, CD117, CD123, CD127, CD235ab/CD61, BDCA3, CCR7, FceRIa, FoxP3, γδTCR, HLA-DR, T-bet, TCR Va24-Ja18, and VISTA. Rphenograph clustering with a k value of 200 yielded 8 clusters, which were annotated based on the relative staining of CD4, CD8, FoxP3, CD25, CD127, CCR7, CD45RA, T-bet, and HLA-DR. Cytobank was used to perform manual gating and create landmark nodes for Statistical Scaffold analysis^35^. Live T cells (Singlets, Intercalator^+^ Cisplatin^−^ CD45^+^, CD61^−^, CD235ab^−^, CD3^+^, CD19^−^) were extracted from the fcs files and divided up into 30 unsupervised clusters using Statistical Scaffold. Clusters were assigned vectors associated with the average median value of markers and edges, which were defined as similarity between vectors to produce graphs, which show the relationships between different clusters. Cluster frequencies and Boolean expression for functional markers for each cluster were passed through the Significance Across Microarrays algorithm, and results were formulated into the Scaffold maps for visualization (github.com/nolanlab/scaffold). For Scaffold Heatmaps, fold-change, significance, cell count, and nearest landmark node data were extracted from the outputs of the Scaffold analysis using a custom script. The heat map was created in R using pheatmap (https://CRAN.R-project.org/package=pheatmap), with log_2_ fold-change capped at 2.

### Multiplex immunofluorescence

Tumor tissue was collected at baseline (pre-treatment) and at C2D15 (or at progression if progression occurred prior to C2D15). Core biopsies or punch biopsies were taken from a lesion that would be injected with T-VEC (e.g., “injected lesion”) as well as from an adjacent non-injected lesion if present (e.g., “non-injected lesion”). Tissues were processed to FFPE blocks, and immune profiling of injected and non-injected lesions was performed using multiplex immunofluorescence as previously described^36^. Sections were stained with two multiplex immune panels containing the following markers – IP1.2: CD3, CD20, Foxp3, pan-cytokeratins, Ki-67, and HLADR; IP2.2: CD3, CD8, CD68, pan-cytokeratins, PD-1, and PD-L1 (Supplementary Table S2). Staining was performed on a Leica BOND Rx, and stained slides were scanned on a Vectra Polaris Imaging System (Akoya Biosciences). Prior to analyses, all images were assessed for quality control. Criteria for rejection included poor tissue quality (e.g., folded tissue or missing sections) or staining artifacts (e.g., air bubbles, signal dropout, or inadequate washing). Cell segmentation and phenotyping were performed in QuPath and Rstudio. The two mIF panels identified 23 cell populations (Supplementary Table S3). To study the spatial distribution of cancer cells and immune cells, each mIF image was virtually divided into non-overlapping hexagons (125 um sides), and the number of cancer cells and immune cells within each hexagon was counted. To calculate colocalization using these cell counts, we applied Morisita-Horn’s (MH) similarity index^37^. The MH index ranges from 0 (highly segregated cell populations) to 1 (highly colocalized). MH indices were calculated for 20 pairs of cell types (Supplementary Table S2).

### Statistical analysis

The primary objective of this study was to assess the safety and efficacy of T-VEC in combination with chemotherapy or endocrine therapy, and to determine the MTV of intratumoral T-VEC that could be administered with gemcitabine and carboplatin (not to exceed 4 mL). The MTV was defined as the highest T-VEC volume (up to 4 mL) that results in ≤1 DLT for patients in the gemcitabine/carboplatin group during cycle 1. Secondary objectives were to evaluate the efficacy of T-VEC in combination with chemotherapy or endocrine therapy using irRECIST 1.1 criteria, including ORR and response duration, and local injection site clinical response. Data were analyzed using Prism Software (GraphPad; San Diego, CA). Descriptive statistics were used to summarize numeric responses as the rate of events (%) and median (range) as appropriate. Wilcoxon rank sum tests were used to evaluate associations between treatment response and immune cell populations/spatial metrics.

## Supplementary information

Supplementary Materials

## Data Availability

Data is provided within the manuscript or supplementary information files. Additional data provided upon reasonable request to the corresponding author.

## References

[1] Cortes, J. et al. Pembrolizumab plus chemotherapy versus placebo plus chemotherapy for previously untreated locally recurrent inoperable or metastatic triple-negative breast cancer (KEYNOTE-355): a randomised, placebo-controlled, double-blind, phase 3 clinical trial. *Lancet Lond. Engl.* **396**, 1817–1828 (2020). 10.1016/S0140-6736(20)32531-9 33278935

[2] Schmid, P. et al. Pembrolizumab for Early Triple-Negative Breast Cancer. *N. Engl. J. Med* **382**, 810–821 (2020). 32101663 10.1056/NEJMoa1910549

[3] Liu, B. L. et al. ICP34.5 deleted herpes simplex virus with enhanced oncolytic, immune stimulating, and anti-tumour properties. *Gene Ther.* **10**, 292–303 (2003). 12595888 10.1038/sj.gt.3301885

[4] Kaufman, H. L., Kohlhapp, F. J. & Zloza, A. Oncolytic viruses: a new class of immunotherapy drugs. *Nat. Rev. Drug Discov.* **14**, 642–662 (2015). 26323545 10.1038/nrd4663 PMC7097180

[5] Andtbacka, R. H. I. et al. Talimogene Laherparepvec Improves Durable Response Rate in Patients With Advanced Melanoma. *J. Clin. Oncol. J. Am. Soc. Clin. Oncol.* **33**, 2780–2788 (2015). 10.1200/JCO.2014.58.3377 26014293

[6] Andtbacka, R. H. I. et al. Final analyses of OPTiM: a randomized phase III trial of talimogene laherparepvec versus granulocyte-macrophage colony-stimulating factor in unresectable stage III–IV melanoma. *J. Immunother. Cancer* **7**, 145 (2019). 31171039 10.1186/s40425-019-0623-z PMC6554874

[7] Kai, M. et al. A phase II study of talimogene laherparepvec for patients with inoperable locoregional recurrence of breast cancer. *Sci. Rep.* **11**, 22242 (2021). 34782633 10.1038/s41598-021-01473-2 PMC8593093

[8] Soliman, H. et al. A Phase I Trial of Talimogene Laherparepvec in Combination with Neoadjuvant Chemotherapy for the Treatment of Nonmetastatic Triple-Negative Breast Cancer. *Clin. Cancer Res J. Am. Assoc. Cancer Res* **27**, 1012–1018 (2021). 10.1158/1078-0432.CCR-20-3105 33219014

[9] Soliman, H. et al. Oncolytic T-VEC virotherapy plus neoadjuvant chemotherapy in nonmetastatic triple-negative breast cancer: a phase 2 trial. *Nat. Med* **29**, 450–457 (2023). 36759673 10.1038/s41591-023-02210-0

[10] Buchanan, C. L. et al. Locoregional recurrence after mastectomy: incidence and outcomes. *J. Am. Coll. Surg.* **203**, 469–474 (2006). 17000389 10.1016/j.jamcollsurg.2006.06.015

[11] Jacobson, J. A. et al. Ten-year results of a comparison of conservation with mastectomy in the treatment of stage I and II breast cancer. *N. Engl. J. Med* **332**, 907–911 (1995). 7877647 10.1056/NEJM199504063321402

[12] Shen, M. C. et al. Clinical course of breast cancer patients with isolated sternal and full-thickness chest wall recurrences treated with and without radical surgery. *Ann. Surg. Oncol.* **20**, 4153–4160 (2013). 23959054 10.1245/s10434-013-3202-4

[13] Zhu, A. et al. Surgical reduction in chest wall disease to prolong survival in breast cancer patients: a retrospective study. *Gland Surg.* **11**, 1015–1025 (2022). 35800744 10.21037/gs-22-246 PMC9253183

[14] Fattahi, S. et al. Reirradiation for Locoregional Recurrent Breast Cancer. *Adv. Radiat. Oncol*. **6**. 10.1016/j.adro.2020.100640 (2021). 10.1016/j.adro.2020.100640 PMC7814100 33506143

[15] Lee, H. K. et al. Superficial hyperthermia and irradiation for recurrent breast carcinoma of the chest wall: prognostic factors in 196 tumors. *Int J. Radiat. Oncol. Biol. Phys.* **40**, 365–375 (1998). 9457823 10.1016/s0360-3016(97)00740-2

[16] Jones, E. L. et al. Randomized trial of hyperthermia and radiation for superficial tumors. *J. Clin. Oncol. J. Am. Soc. Clin. Oncol.* **23**, 3079–3085 (2005). 10.1200/JCO.2005.05.520 15860867

[17] Saunders, Y., Stebbing, J., Broadley, K. & Johnston, S. R. D. Recurrent Locally Advanced Breast Cancer: The Treatment of Chest Wall Disease with Further Chemotherapy. *Clin. Oncol.* **13**, 195–199 (2001). 10.1053/clon.2001.9252 11527294

[18] Salazar, L. G. et al. Topical Imiquimod Plus Nab-paclitaxel for Breast Cancer Cutaneous Metastases: A Phase 2 Clinical Trial. *JAMA Oncol.* **3**, 969–973 (2017). 28114604 10.1001/jamaoncol.2016.6007 PMC5824239

[19] Vidula, N. et al. Multi-center randomized study of pembrolizumab/carboplatin versus carboplatin alone in patients with chest wall disease from breast cancer: TBCRC 044. *J. Clin. Oncol.* **39**, TPS1111–TPS1111 (2021).

[20] Cardoso, F., McArthur, H., Schmidt, P. & Cortes, J. KEYNOTE-756: Phase 3 Study of Neoadjuvant Pembrolizumab or Placebo + Chemotherapy Followed by Adjuvant Pembrolizumab or Placebo _+ Endocrine Therapy for Early-Stage High-Risk ER+/HER2– Breast Cancer. Presented at: October 20, Madrid, Spain. (2023).

[21] Loi, S., Curigliano, G. & Salgado, R. Biomaker results in high-risk estrogen receptor-positive, human epidermal growth factor receptor 2-negative primary breast cancer following neoadjvuant chemotherpay +/− nivolumab: an exploratory analysis of CheckMate 7FL. Presented at: SABCS; December 6, San Antonio, TX. (2023).

[22] Hamid, O., Ismail, R. & Puzanov, I. Intratumoral Immunotherapy-Update 2019. *Oncologist* **25**, e423–e438 (2020). 32162802 10.1634/theoncologist.2019-0438 PMC7066689

[23] Huppert, L. A. & Daud, A. I. Intratumoral therapies and in-situ vaccination for melanoma. *Hum. Vaccines Immunother.* **18**, 1890512 (2022). 10.1080/21645515.2021.1890512 PMC9116417 35559766

[24] Lutzky, J. et al. Optimization of Voyager V1 (VV1) oncolytic virus systemic delivery in combination with cemiplimab and ipilimumab in patients with melanoma and non–small cell lung cancer (NSCLC). *J. Clin. Oncol.* **40**, TPS9595–TPS9595 (2022).

[25] Chien, A. J. et al. Evaluation of intra-tumoral (IT) SD-101 and pembrolizumab (Pb) in combination with paclitaxel (P) followed by AC in high-risk HER2-negative (HER2-) stage II/III breast cancer: Results from the I-SPY 2 trial. *J. Clin. Oncol.* **39**, 508–508 (2021).

[26] Jacob, S. et al. Regional lymph node changes on breast MRI in patients with early-stage breast cancer receiving neoadjuvant chemo-immunotherapy. *Breast Cancer Res Treat*. Published online September 21, 10.1007/s10549-024-07481-w (2024). 10.1007/s10549-024-07481-w PMC11785630 39305392

[27] Runcie, K. et al. Phase I study of intratumoral injection of talimogene laherparepvec for the treatment of advanced pancreatic cancer. *Oncologist* **30**, oyae200 (2025). 39673447 10.1093/oncolo/oyae200 PMC11783283

[28] Jo, H. H. et al. Early Experience of Oncolytic Virus Injection Combined with Sorafenib in a Patient with Advanced Hepatocellular Carcinoma and Portal Vein Thrombosis. *J. Liver Cancer* **20**, 177–182 (2020). 37384323 10.17998/jlc.20.2.177 PMC10035676

[29] Kaufman, H. L. et al. Local and distant immunity induced by intralesional vaccination with an oncolytic herpes virus encoding GM-CSF in patients with stage IIIc and IV melanoma. *Ann. Surg. Oncol.* **17**, 718–730 (2010). 19915919 10.1245/s10434-009-0809-6

[30] Moesta, A. K. et al. Local Delivery of OncoVEXmGM-CSF Generates Systemic Antitumor Immune Responses Enhanced by Cytotoxic T-Lymphocyte-Associated Protein Blockade. *Clin. Cancer Res J. Am. Assoc. Cancer Res* **23**, 6190–6202 (2017). 10.1158/1078-0432.CCR-17-0681 28706012

[31] Li, S. et al. Predictive and prognostic values of tumor infiltrating lymphocytes in breast cancers treated with neoadjuvant chemotherapy: A meta-analysis. *Breast J. Eur. Soc. Mastology* **66**, 97–109 (2022). 10.1016/j.breast.2022.10.001 PMC9550538 36219945

[32] Miyashita, M. et al. Tumor-infiltrating CD8+ and FOXP3+ lymphocytes in triple-negative breast cancer: its correlation with pathological complete response to neoadjuvant chemotherapy. *Breast Cancer Res Treat.* **148**, 525–534 (2014). 25395319 10.1007/s10549-014-3197-y

[33] Kotecha, N., Krutzik, P. O. & Irish, J. M. Web-based analysis and publication of flow cytometry experiments. *Curr Protoc Cytom.* Chapter 10:Unit10.17. 10.1002/0471142956.cy1017s53 (2010). 10.1002/0471142956.cy1017s53 PMC4208272 20578106

[34] Levine, J. H. et al. Data-Driven Phenotypic Dissection of AML Reveals Progenitor-like Cells that Correlate with Prognosis. *Cell* **162**, 184–197 (2015). 26095251 10.1016/j.cell.2015.05.047 PMC4508757

[35] Spitzer, M. H. et al. Systemic Immunity Is Required for Effective Cancer Immunotherapy. *Cell* **168**, 487–502.e15 (2017). 28111070 10.1016/j.cell.2016.12.022 PMC5312823

[36] Mori, H. et al. Characterizing the Tumor Immune Microenvironment with Tyramide-Based Multiplex Immunofluorescence. *J. Mammary Gland Biol. Neoplasia* **25**, 417–432 (2020). 33590360 10.1007/s10911-021-09479-2 PMC7960613

[37] Horn, H. S. Measurement of “Overlap” in Comparative Ecological Studies. *Am. Nat.* **100**, 419–424 (1966).

