# Supplementary material for: Phase Ib study of intratumoral talimogene laherparepvec (T-VEC) in combination with chemotherapy or endocrine therapy in patients with advanced HER2-negative breast cancer
Source: NPJ Breast Cancer. 2025 Nov 21;11:130. doi: 10.1038/s41523-025-00842-8 (PMC12638943; doi:10.1038/s41523-025-00842-8)
Supplement: Supplementary file 1 — Supplementary Materials [file 41523_2025_842_MOESM1_ESM.pdf]

Supplementary Materials

Supplementary Figure S1. Study schema

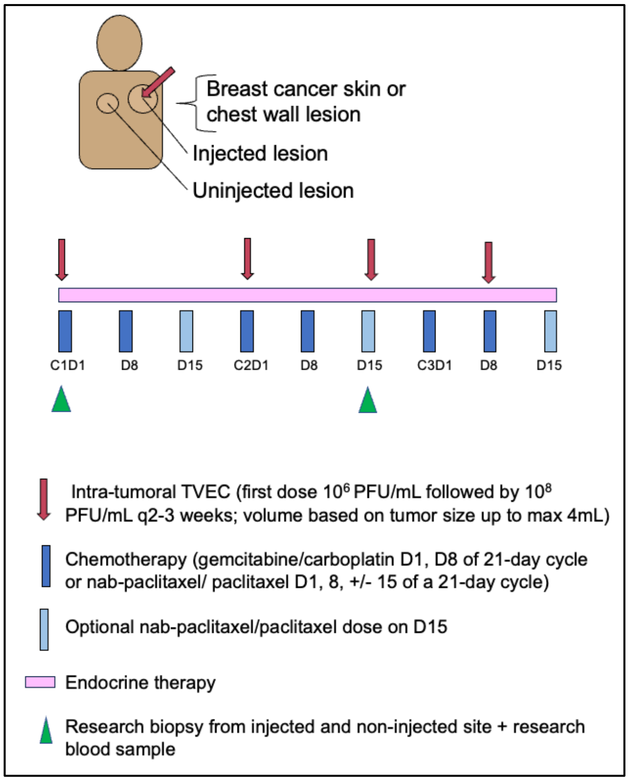

This diagram depicts the study schema.

Supplemental Figure S2: Representative photographs of injected lesion

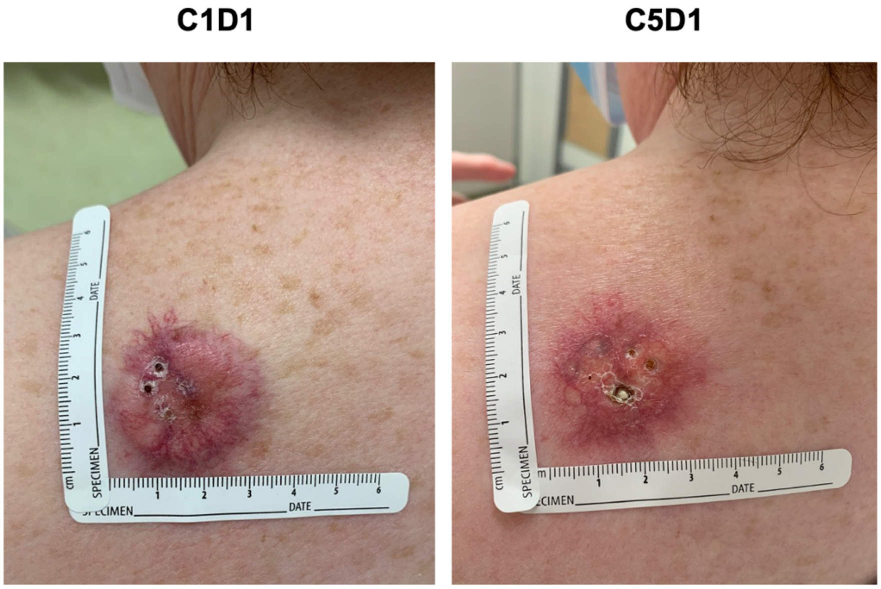

Shown are photographs of an injected lesion from PID-09 on C1D1 (left) and C5D1 (right).

Supplementary Figure S3: Flow cytometry analysis

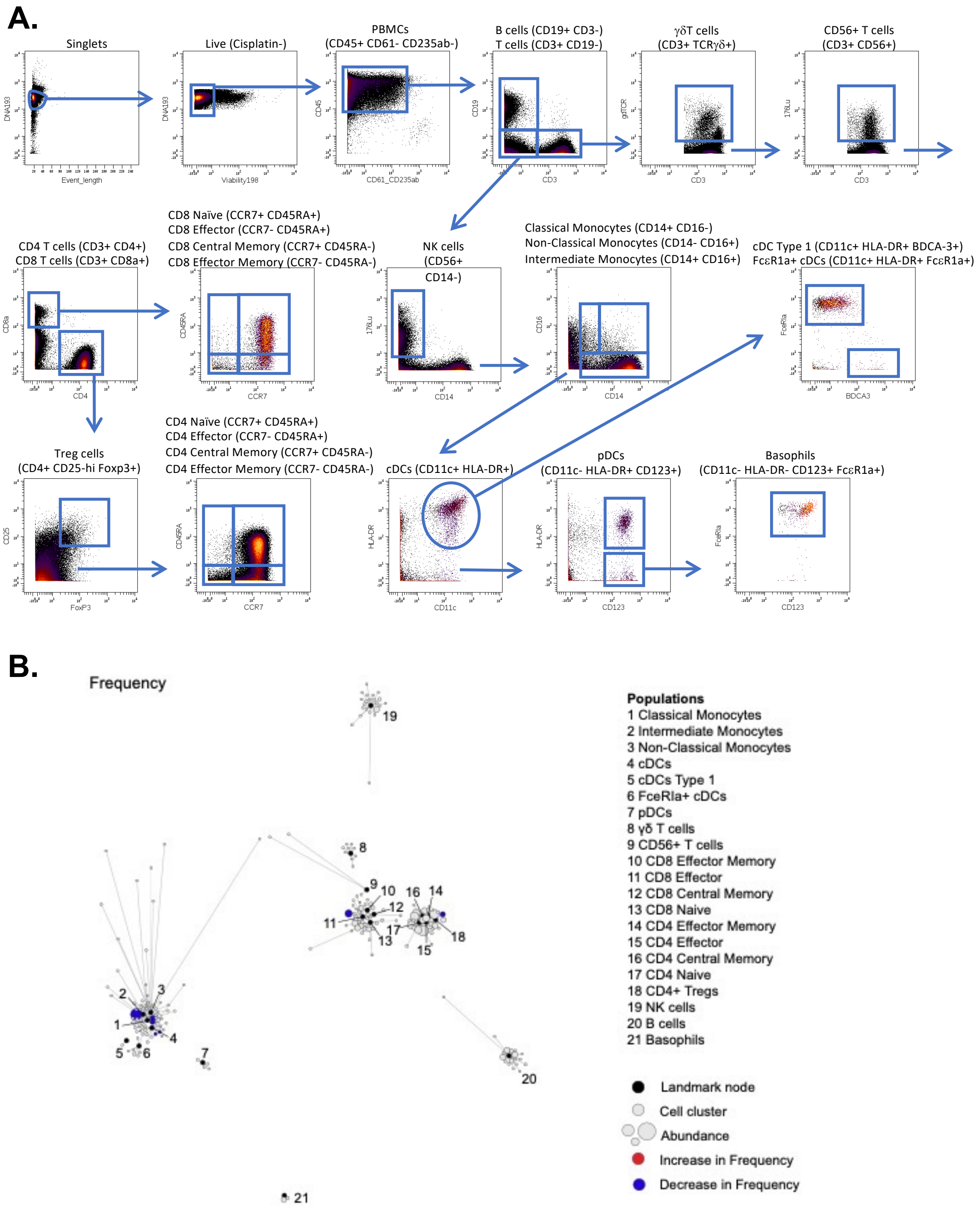

A) The gating strategy for mass cytometry annotation is shown. B) Statistical scaffold analysis was performed to compare baseline vs C2D15 timepoints. Black dots represent landmark nodes. Red dots represent clusters that significantly increase in frequency. Blue dots represent clusters that significantly decrease in frequency.

Supplementary Figure S4: Differences in circulating immune cells

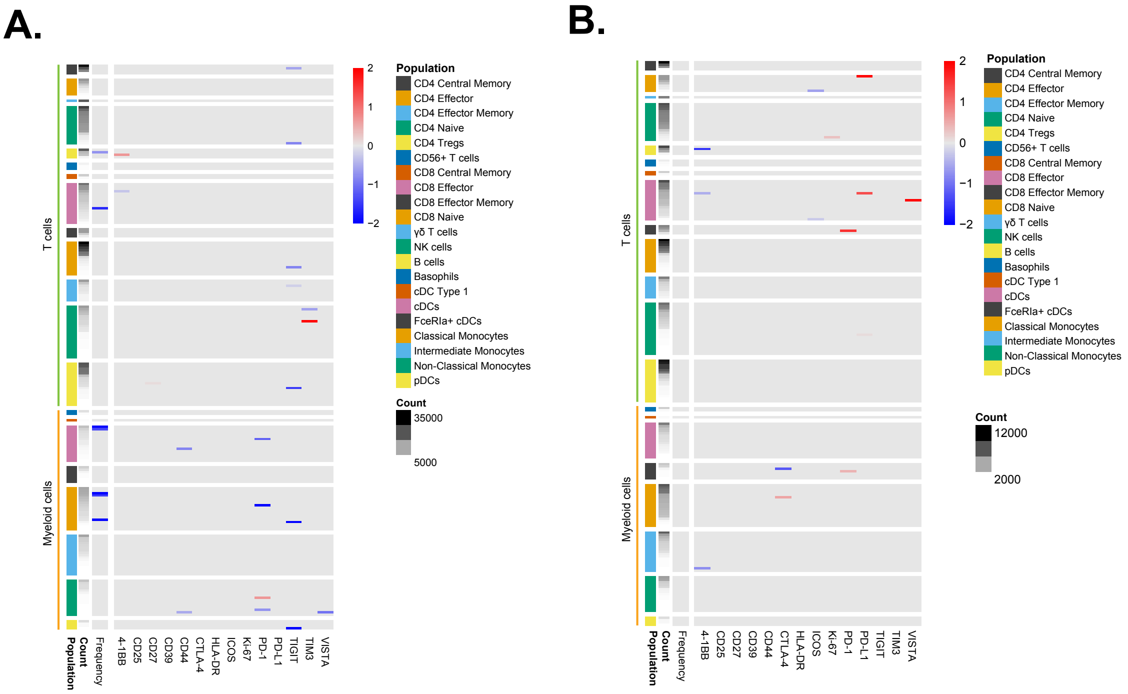

A) Heatmaps summarizing $\log_2$ fold changes resulting from statistical scaffold analysis of cell cluster frequency (left most column and functional markers (4-1BB, CD25, CD27, CD39, CTLA-4, HLA-DR, ICOS, Ki-67, PD-1, PD-L1, TIGIT, TIM3 and VISTA) are shown comparing baseline vs. C2D15. Red denotes an increase in the post-treatment timepoint; blue denotes a decrease in the post-treatment timepoint. B) Heatmap showing differences from statistical scaffold analysis in cellular frequencies (left column) and phenotypic markers comparing responders vs. responders at the pre-treatment timepoint. Red denotes an increase in the responders; blue denotes a decrease in the responders.

Supplementary Figure S5: Induction of Ki-67 on different myeloid populations

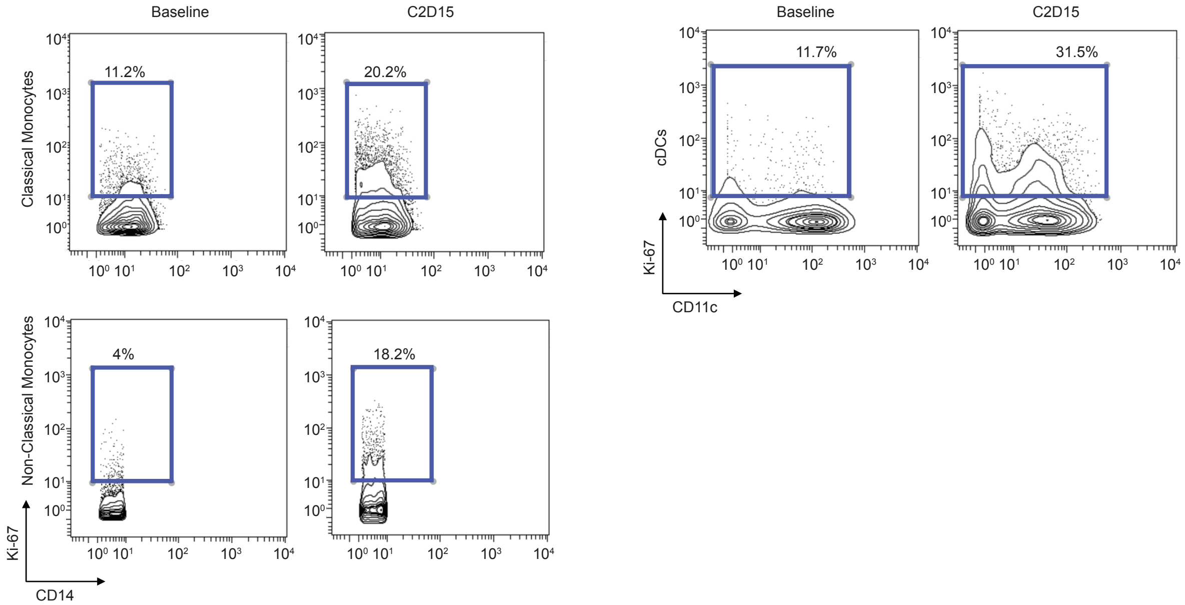

The indicated myeloid populations were gated on from a clinical responder with Ki-67 (y-axis) shown at baseline and C2D15. Results are representative of responders.

Supplementary Figure S6: Example mIF images from a patient with low TIL and high TIL

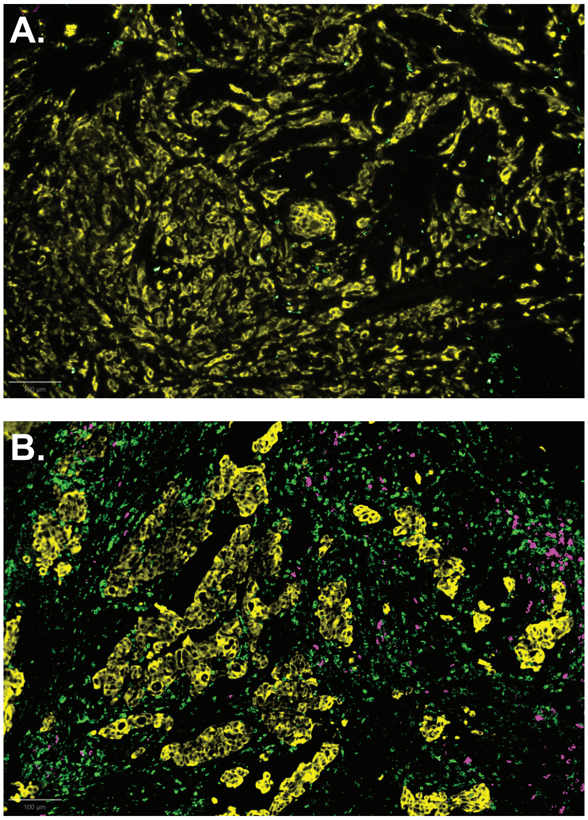

Example images of pre-treatment biopsies stained with mIF Panel IP1.2 from a patient with low TIL counts (A) and high TIL counts (B). Yellow: CK^+ tumor cells; Green: $CD3^+$ T cells; Magenta: $CD20^+$ B cells.

Supplemental Table S1: Heavy metal-labelled antibodies for analysis by mass cytometry by time-of-flight (CyTOF)

#	Metal tag	Target	Clone	Catalog #	Vendor	Lot #
1	89	CD45	HI30	3089003B	Fluidigm	1831803
2	113	CD61	V1-PL2	336402	Biolegend	B176208 - 4/13/2018
3	113	CD235ab	HIR2	306602	Biolegend	B187326 - 4/13/2018
4	115	CD71 (Transferrin receptor)	OKT9	14-0719-82	Invitrogen	1986927 - 11/28/2018
5	139	CD11c	Bu15	337202	Biolegend	B241075 - 7/10/2018
6	140	CD15	W6D3	323002	BioLegend	B254011 - 8/18/2018
7	141	CD3	UCHT1	3141019B	Fluidigm	2501812
8	142	CD19	HIB19	3142001B	Fluidigm	171815
9	143	CD117	104D2	3143001B	Fluidigm	1351805
10	144	CD11b	ICRF44	3144001B	Fluidigm	241824
11	145	CD4	RPA-T4	3145001B	Fluidigm	3391711
12	146	CD8a	RPA-T8	3146001B	Fluidigm	1201824
13	147	OX40	Ber-ACT35	350002	BioLegend	B247739 - 8/31/2018
14	148	CD14	RMO52	3148010B	Fluidigm	2501814
15	149	CD127	A019D5	3149011B	Fluidigm	1921820
16	150	FceRIa	AER-37 (CRA-1)	3150027B	Fluidigm	2821705
17	151	CD123	6H6	3151001B	Fluidigm	671815
18	152	TCRγδ	B1	331202	Biolegend	B232819 - 8/2/2018
19	153	CD45RA	HI100	3153001B	Fluidigm	1921817
20	154	TIM3	F38-2E2	3154010B	Fluidigm	1911813
21	155	TIGIT	MBSA43	16-9500-82	eBiosciencee	4305630 - 8/2/2018
22	156	PD-L1 (CD274)	29E.2A3	3156026B	Fluidigm	371818
23	158	CD27	L128	3158010B	Fluidigm	741808
24	159	CD137 (4-1BB)	4B4-1	B227357	Biolegend	B227357 - 4/18/2018
25	160	Tbet	4B10	3160010B	Fluidigm	671822
26	161	CD152 (CTLA-4)	14D3	3161004B	Fluidigm	2651711
27	162	FoxP3	259D/C7	3162024A	Fluidigm	1651813
28	163	CD31	WM53	303108	Biolegend	B176236 - 10/9/2017
29	164	CD95 (FAS)	DX2	3164008B	Fluidigm	3391720
30	165	VISTA-PE	D1L2G	18946S	Cell Signaling Technologies	
31	165	PE	PE001	3165015B	Fluidigm	801827
32	166	NKG2D	ON72	3166016B	Fluidigm	2511702
33	167	CCR7 (CD197)	G043H7	3167009A	Fluidigm	2201822
34	168	Ki-67	B56	3168007B	Fluidigm	581816
35	169	CD25	2A3	3169003B	Fluidigm	1801806
36	170	CD154 (CD40L)	24-31	310802	BioLegend	B185875 - 11/28/2018
37	171	CD40	5C3	334302	BioLegend	B255767 - 11/28/2018
38	172	PD-L2	24F.10C12	3172014B	Fluidigm	151804
39	173	HLA-DR	L243	3173005B	Fluidigm	(titrated lot: 3331706 HLA-DR 174) 671820
40	174	PD-1	EH12.2H7	3174020B	Fluidigm	(titrated lot: 0371819 PD-1 175) 1711703
41	175	ICOS	C398.48	313502	Biolegend	B222851 - 7/2/2018
42	176	CD56	NCAM16.2	3176008B	Fluidigm	1671712
43	209	CD16	3G8	3209002B	Fluidigm	1521806

Blue shading = intracellular stain. Green = pre-stain

Supplemental Table S2: Details of primary antibodies and Opal fluorophores used in mIF

**mIF Panel IP1.2**			
Marker	***Clone***	***Antibody source***	***Fluorophore***
FoxP3; regulatory T cell marker	236A/E7	Abcam	Opal570
pan-cytokeratin; epithelial cell marker	AE1/AE3	Dako	Opal690
CD20; B lymphocyte marker	L26	Ventana/Roche	Opal520
CD3; T lymphocyte marker	2GV6	Ventana/Roche	Opal480
Ki67; proliferation marker	30-9	Ventana/Roche	Opal620
HLA-DR; MHC class II marker	EPR3692	Abcam	AlexaFluor750
**mIF Panel IP2.2**			
Marker	***Clone***	***Antibody source***	***Fluorophore***
PD-L1; immune checkpoint	E1L3N	Cell Signaling	Opal520
PD-1; immune checkpoint	EPR4877	Abcam	Opal620
CD8; cytotoxic T cell marker	4B11	Leica	Opal570
pan-cytokeratin; epithelial cell marker	AE1/AE3	Dako	Opal690
CD68; macrophage marker	PG-M1	Dako	AlexaFluor750
CD3; T cell marker	2GV6	Ventana/Roche	Opal480

Supplemental Table S3: Immune biomarkers evaluated in this study via mIF

Biomarker	Description
TIL	Tumor infiltrating lymphocytes (T cells and B cells)
Tcell	T cells (CD3+)
Bcell	B cells (CD20+)
Tc	Cytotoxic T cells (CD8+CD3+)
CD8nT	CD8-negative T cells (CD8-CD3+)
Treg	Regulatory T cells (Foxp3+CD3+)
Mac	Macrophages (CD68+CK-)
Ki67pTIL	proliferating TIL (Ki67+ T cells and Ki67+ B cells)
Ki67pT	proliferating T cells (Ki67+CD3+)
Ki67pTreg	proliferating Treg cells (Ki67+Foxp3+CD3+)
Ki67pB	proliferating B cells (Ki67+CD20+)
Ki67pTum	proliferating tumor cells (Ki67+CK+)
HLADRpTIL	MHC ClassII positive TIL (HLADR+ T cells and HLADR+ B cells)
HLADRpT	MHC ClassII positive T cells (HLADR+ CD3+)
HLADRpB	MHC ClassII positive B cells (HLADR+ CD20+)
HLADRpTreg	MHC ClassII positive Treg cells (HLADR+Foxp3+CD3+)
HLADRpTum	MHC ClassII positive tumor cells (HLADR+ CK+)
PD1pT	PD-1 positive T cells (PD-1+ CD3+)
PD1pTc	PD-1 positive cytotoxic T cells (PD-1+ CD8+ CD3+)
PD1pCD8nT	PD-1 positive CD8 negative T cells (PD-1+ CD8- CD3+)
PDL1pMac	PD-L1 positive macrophages (PD-L1+ CD68+ CK-)
PDL1pTum	PD-L1 positive tumor cells (PD-L1+ CK+)
AnyPDL1	Any PD-L1 positive cell
T_B.MHI	**Morisita-Horn Index**<br>cellA_cellB.MHI: colocalization of cell type A & cell type B<br>example: TIL_Tum.MHI - colocalization of TIL and tumor cells
T_Mac.MHI	
Tc_CD8nT.MHI	
Tc_Mac.MHI	
CD8nT_Mac.MHI	
Treg_Foxp3nT.MHI	
Treg_B.MHI	
TIL_Tum.MHI	
T_Tum.MHI	
Tc_Tum.MHI	
CD8nT_Tum.MHI	
Treg_Tum.MHI	
B_Tum.MHI	
Mac_Tum.MHI	
PDL1Mac_PD1T.MHI	
PDL1Mac_PD1pTc.MHI	
PDL1Mac_PD1pCD8nT.MHI	
PDL1Tum_PD1T.MHI	
PDL1Tum_PD1pTc.MHI	
PDL1Tum_PD1pCD8nT.MHI	

Supplemental Tables 4. Treatment-related adverse events by treatment arm

Table S4A. Treatment-related adverse events: Endocrine Therapy + TVEC

Treatment-related adverse event (n=2)	Any grade, n (%)	Grade 3-4, n (%)
Injection site pain	1 (50%)	0 (0%)
Injection site skin ulceration	1 (50%)	0 (0%)
Chills	1 (50%)	0 (0%)
Fever	1 (50%)	0 (0%)
Body aches / myalgias	1 (50%)	0 (0%)
Fatigue	1 (50%)	0 (0%)

Table S4B Treatment-related adverse events Paclitaxel/Nab-paclitaxel + TVEC

Treatment-related adverse event (n=9)	Any grade, n (%)	Grade 3-4, n (%)
Injection site pain	4 (44.4%)	0 (0%)
Injection site erythema/redness	1 (11.1%)	0 (0%)
Injection site skin ulceration	2 (22.2%)	1 (11.1%)
Chills	4 (44.4%)	0 (0%)
Fever	7 (77.8%)	0 (0%)
Body aches / myalgias	2 (22.2%)	0 (0%)
Fatigue	6 (66.7%)	0 (0%)
Nausea	4 (44.4%)	0 (0%)
Emesis	2 (22.2%)	0 (0%)
Anorexia	3 (33.3%)	0 (0%)
Neuropathy	5 (55.6%)	0 (0%)
Dysgeusia	2 (22.2%)	0 (0%)
Diarrhea	1 (11.1%)	0 (0%)
Infusion reaction	2 (22.2%)	0 (0%)
Anemia	2 (22.2%)	0 (0%)
Thrombocytopenia	1 (11.1%)	0 (0%)
Neutrophil count decreased	2 (22.2%)	2 (55.6%)

Table S4C Treatment-related adverse events: Gemcitabine + Carboplatin + TVEC

Treatment-related adverse event (n=8)	Any grade, n (%)	Grade 3-4, n (%)
Injection site pain	2 (25.0%)	0 (0%)
Injection site erythema/redness	1 (12.5%)	0 (0%)
Injection site skin ulceration	1 (12.5%)	0 (0%)
Chills	3 (37.5%)	0 (0%)
Fever	6 (75.0%)	0 (0%)
Body aches / myalgias	1 (12.5%)	0 (0%)
Fatigue	7 (87.5%)	0 (0%)
Nausea	6 (75.0%)	0 (0%)
Emesis	1 (12.5%)	0 (0%)
Anorexia	1 (12.5%)	0 (0%)
Dysgeusia	2 (25.0%)	0 (0%)
Diarrhea	2 (25.0%)	0 (0%)
Infusion reaction	1 (12.5%)	0 (0%)
Anemia	1 (12.5%)	1 (12.5%)
Thrombocytopenia	5 (62.5%)	2 (25.0%)
Neutrophil count decreased	5 (62.5%)	4 (50.0%)

Shown are the most frequent treatment-related adverse events by CTCAE v 4.0, including any treatment-related adverse event present in >10% patients and/or any grade 3 or greater event. No grade 5 events occurred.
Abbreviation: n, number

Supplemental Table S5. Individual patient characteristics, treatment, and responses

PID	Receptor status	PDL1 status (testing method) for pts w mTNBC	Total prior lines tx for MBC (prior chemo)	Systemic treatment partner	Max T-VEC volume injected per visit	# T-VEC injections	Site injected	Time on study (weeks)	Best response per irRECIST	Best response at site of local T-VEC injection
**04**	**HR+/HER2-** ER 80% PR: 0%		0 (0 chemo)	P → Nab-P	4 mL	8	Fungating breast/chest wall mass	23.0	PR	PR
**09**	**HR+/HER2-** ER: 95% PR: 95%		4 (2 chemo)	Nab-P	4 mL	14	Subcutaneous nodule	43.9	PR	PR
**11**	**HR+/HER2-** ER: 30% PR: 2%		5 (2 chemo)	G/C	4 mL	10	Non-fungating breast mass	27.1	SD	SD
**06**	**TNBC**	Pos. (PD-L1 SP142 5% IC intensity 3+)	3 (2 chemo)	Nab-P	4 mL	7	Non-fungating breast mass	17.1	SD	SD
**10**	**TNBC**	Neg. (PD-L1 SP142 TPS 0%)	1 (0 chemo)	G/C	4 mL	9	Subcutaneous nodule	34.9	SD	PR
**14**	**HR+/HER2-** ER: 90% PR: 10%		4 (1 chemo)	P	4 mL	3*	Fungating breast/chest wall mass	12.1	SD	SD
**20**	**TNBC**	Neg. (PD-L1 22C3 CPS 0)	4 (2 chemo)	G/C	2 mL	2*	Fungating breast/chest wall mass	33.9	SD	PR
**13**	**TNBC**	Pos. (PD-L1 22C3 CPS 11-20)	2 (2 chemo)	Nab-P	4 mL	7**	Fungating breast/chest wall mass	17.9	SD	PR
**15**	**TNBC**	Not tested	2 (2 chemo)	G/C	4 mL	4	Non-fungating breast mass	8.9	SD	PR
**01**	**HR+/HER-** ER >95% PR 0%		5 (3 chemo)	Tamoxifen	1 mL	2*	Fungating breast/chest wall mass	6.7	PD	SD
**02**	**TNBC**	Not tested	5 (2 chemo)	G/C	4 mL	3	Fungating breast/chest wall mass	12.0	PD	SD
**05**	**TNBC**	Neg. per clinical notes (report not available)	1 (1 chemo)	Nab-P	2 mL	4	Non-fungating breast mass	8.4	PD	SD
**07**	**TNBC**	Pos. (PD-L1 SP142 IC >/=1%)	2 (2 chemo)	Nab-P	2 mL	4	Subcutaneous nodule	8.9	PD	PD
**16**	**TNBC**	Pos. (PD-L1 SP142: 1%)	4 (4 chemo)	G/C	4 mL	4	Subcutaneous nodule	8.9	PD	PR
**17**	**HR+/HER2-** ER: >90% PR: 0%		8 (4 chemo)	G/C	2 mL	4	Non-fungating breast mass	10.9	PD	PR
**21**	**TNBC**	Neg. (PD-L1 CPS 0)	1 (1 chemo)	P	4 mL	4	Non-fungating breast mass	8.1	PD	SD
**08**	**HR+/HER2-** ER: 11-20% PR: 0%		8 (6 chemo)	Nab-P	1.5 mL	1	Fungating breast/chest wall mass	1.0	n/a	n/a

**18**	**HR+/HER2-** ER: 30% PR: <1%		2 (1 chemo)	G/C	2 mL	1	Non-fungating breast mass	3.0	n/a	PD
**19**	**HR+/HER2-** ER: 0% PR: ~55%		4 (1 chemo)	Fulvestrant	2 mL	1	Subcutaneous nodule	2.3	n/a	PD

* T-VEC stopped early due to local site ulceration; systemic therapy continued until progression.

** T-VEC stopped due to ulceration but then restarted at a different injection site.

Abbreviations: HR+/HER2-, hormone-receptor positive, human epidermal growth factor receptor-2 negative; TNBC, triple negative breast cancer; P, paclitaxel; Nab-P, nab -paclitaxel; G/C, gemcitabine/carboplatin; R, right; L, left; n/a, not available
